# The Cognitive and Behavioural Impact of Alcohol Promoting and Alcohol Warning Advertisements: An Experimental Study

**DOI:** 10.1093/alcalc/agv104

**Published:** 2015-09-20

**Authors:** Kyle G. Brown, Kaidy Stautz, Gareth J. Hollands, Eleanor M. Winpenny, Theresa M. Marteau

**Affiliations:** 1 Behaviour andHealth Research Unit, University of Cambridge, Cambridge, UK; 2 RAND Europe, Westbrook Centre, Cambridge, UK

## Abstract

**Aims:**

To assess the immediate effect of alcohol promoting and alcohol warning advertisements on implicit and explicit attitudes towards alcohol and on alcohol seeking behaviour.

**Methods:**

We conducted a between-participants online experiment in which participants were randomly assigned to view one of three sets of advertisements: (a) alcohol promoting, (b) alcohol warning, or (c) unrelated to alcohol. A total of 373 participants (59.5% female) aged 18–40 (*M* = 28.03) living in the UK were recruited online through a research agency. Positive and negative implicit attitudes and explicit attitudes towards alcohol were assessed before and after advertisements were viewed. Alcohol seeking behaviour was measured by participants' choice of either an alcohol-related or non-alcohol-related voucher offered ostensibly as a reward for participation. Self-reported past week alcohol consumption was also recorded.

**Results:**

There were no main effects on any of the outcome measures. In heavier drinkers, viewing alcohol promoting advertisements increased positive implicit attitudes (standardized beta = 0.15, *P* = 0.04) and decreased negative implicit attitudes (standardized beta = −0.17, *P* = 0.02). In heavier drinkers, viewing alcohol warning advertisements decreased negative implicit attitudes (standardized beta = −0.19, *P* = 0.01).

**Conclusions:**

Viewing alcohol promoting advertisements has a cognitive impact on heavier drinkers, increasing positive and reducing negative implicit attitudes towards alcohol. Viewing alcohol warning advertisements reduces negative implicit attitudes towards alcohol in heavier drinkers, suggestive of a reactance effect.

## INTRODUCTION

Alcohol promoting messages occur in 20% of television advertisement breaks during prime-time broadcasting hours in the UK (Lyons *et al.*, 2014). Alcohol advertising is also prevalent in the USA, where 91% of those aged 12–20 are exposed to televised alcohol advertising (Centre on Alcohol Marketing and Youth, 2010), and in Australia, where adolescents under the legal drinking age are as likely as young adults to be exposed to alcohol advertisements (Winter *et al.*, 2008; Pettigrew *et al.*, 2012). In an effort to counter the pervasive nature of alcohol advertising, governments and public health organizations have developed alcohol use warning advertisements that highlight the dangers of alcohol consumption and encourage responsible use.

There is now a reasonable level of evidence from observational data that exposure to alcohol promoting advertising is associated with future alcohol use (Anderson *et al.*, 2009; Smith and Foxcroft, 2009). Furthermore, a recent systematic review and meta-analysis of experimental studies investigating the immediate effects of alcohol promoting advertising on alcohol consumption found evidence that a one-off exposure to alcohol promoting advertising may increase amounts of alcoholic beverage consumed following exposure by small amounts, equivalent to between 0.39 and 2.85 alcohol units for males and between 0.25 and 1.81 units for females (K Stautz *et al.*, in preparation). These studies were limited, however, by a sole focus on undergraduate students as participants and inadequate power to detect small effects. Regarding alcohol warning advertisements, whilst there is a small amount of observational evidence supporting the effectiveness of these messages in relation to reductions in drink-driving (Agostinelli and Grube, 2002), there is a dearth of experimental studies that test their immediate effects.

In examining whether alcohol promoting and alcohol warning advertisements influence alcohol use, it is important to consider potential mechanisms of effect. One way in which advertising may affect behaviour is through changes in implicit and explicit attitudes towards alcohol. Implicit attitudes are memory associations outside of conscious awareness that can be activated automatically (Greenwald and Banaji, 1995), whereas explicit attitudes comprise individuals' consciously reported beliefs. Implicit and explicit attitudes towards alcohol are modestly related, and both account for unique variance in measures of alcohol consumption (Reich *et al.*, 2011).

Longitudinal evidence suggests an indirect effect of alcohol promoting advertising on alcohol use via an increase in positive alcohol-related attitudes (Morgenstern *et al.*, 2011). To our knowledge, only one study provides experimental evidence for such an effect (Goodall and Slater, 2010). Participants in this study were randomized to watch alcohol promoting advertisements, alcohol warning advertisements, or control non-alcohol advertisements. Viewing alcohol promoting advertisements resulted in more positive implicit responding for alcohol compared with viewing alcohol warning or non-alcohol advertisements. Although explicit attitudes towards alcohol were more positive for those in the alcohol promoting relative to the alcohol warning condition, the attitudes of individuals in both conditions did not differ from those in the control group. In addition, both implicit and explicit positive attitudes predicted willingness to engage in risky alcohol-related behaviour.

Goodall and Slater's study does, however, have three design limitations. First, the measure of implicit attitudes used, the Affect Misattribution Procedure, assesses only positive versus negative attitudes. Attitudes towards alcohol are often ambivalent, i.e. comprise both positive and negative attitudes (de Visser and Smith, 2007). The use of a unidimensional measure of implicit attitudes renders it unclear whether the ineffectiveness of the alcohol warning advertisements in Goodall and Slater's study was due to the advertisements' inability to activate negative implicit attitudes, or whether they served as a cue to activate positive implicit attitudes. A second limitation was that the alcohol warning advertisements used focused on drink-driving, which may have been too narrow a focus to change more general attitudes towards alcohol. Third, baseline measures of attitudes towards alcohol were not recorded. Each of these limitations are addressed in the current study: first, by using a measure of implicit attitudes that assesses positive and negative associations separately (an adapted version of the Implicit Association Test; Greenwald *et al.*, 1998; Houben *et al.*, 2010); second, by using alcohol warning advertisements that focus on general negative consequences from alcohol use; and third, by assessing attitudes both pre- and post-exposure to advertisements.

Any impact of alcohol-related advertising on cognition or behaviour may be dependent on individual characteristics. Primarily, an individual's previous experience with alcohol is likely to influence their response to messages that aim to promote or to dissuade alcohol use. Heavier drinkers show increased attention towards alcohol-related cues (Field and Cox, 2008) and may be more likely to attend to alcohol-related advertising and thus be influenced by its message. Education, a marker of socioeconomic status, is another possible effect modifier. Lower levels of education are associated with increased quantity of alcohol consumed per occasion (Casswell *et al.*, 2003; Huckle*et al.*, 2010), and increased likelihood of binge drinking in males (Jefferis *et al.*, 2007). Individual differences in inhibitory control may also be important. Poor inhibitory control has been shown to strengthen the association between implicit attitudes and drinking behaviour (Houben and Wiers, 2009).

### Aims and hypotheses

This study aims to replicate and extend findings by Goodall and Slater (2010) by testing effects of alcohol promoting and alcohol warning advertising on changes in implicit and explicit attitudes and on alcohol seeking behaviour, with typical alcohol consumption and level of education as potential moderators. We predict that individuals exposed to alcohol promoting advertisements, compared with those shown advertisements unrelated to alcohol, will exhibit more positive implicit and explicit attitudes, and be more likely to select an alcohol-related reward. Individuals exposed to alcohol warning advertisements, compared with those shown non-alcohol advertisements, will exhibit more negative implicit and explicit attitudes and be less likely to select an alcohol-related reward. These effects are expected to be increased in heavier drinkers and in those with lower levels of education. Further, we predict that the impact of alcohol promoting and alcohol warning advertisements on choice of reward will be mediated by changes in implicit attitudes. If such a mediation effect is present, we predict that it will be weaker in those with greater inhibitory control.

## MATERIALS AND METHODS

### Participants

A total of 373 participants (59.5% (222) female; Mean age = 28.03, SD = 5.64) completed the study. All participants were based in the UK and were predominantly of White/British ethnic origin (84.5%). Participants were recruited using an agency's existing online survey panel (www.OnePoll.com). A power calculation indicated that 384 participants were required to achieve 80% power to detect all predicted effects, assuming a moderate effect size (equivalent to *f* = 0.25). 540 participants were sampled to allow for around 35% attrition.

### Design

The study used a between-participants experimental design with random allocation to one of three types of advertisement exposure: (a) alcohol promoting advertisements, (b) alcohol warning advertisements or (c) control advertisements unrelated to alcohol.

### Procedure

The study protocol received approval from the University of Cambridge Psychology Department ethics committee (Application No: Pre.2013.73). The study was conducted online using specialized survey (www.qualtrics.com) and experimental software (Inquisit – www.millisecond.com). At initial assessment, participants completed a consent form, demographic questions, and measures of alcohol consumption, implicit and explicit attitudes towards alcohol, and inhibitory control. Participants not meeting the inclusion criteria of being aged 18–40 and residing in the UK were excluded at this point. Eligible participants were sent a web link to complete the remainder of the study. For the second part of the study participants were randomly assigned to experimental condition according to an algorithm used within the survey software. Participants each viewed eight advertisements, presented in random order, for a total duration of between 310 and 330 s. Four advertisements were related to experimental condition whilst four were shown to all groups and served as filler advertisements. Following advertisement presentation participants again completed measures of implicit and explicit attitudes as well as questions on their familiarity with the advertisements and associated brands. They were then asked to select a choice of voucher reward ostensibly as compensation for their time (see Measures). Upon completion participants were debriefed and given £2 worth of credit via the research agency. Participants did not receive their voucher reward, but instead were given an additional £5 worth of credit. (Participants' bank details were held by the research agency irrespective of our study. The agency also offers cheques for participants who do not wish to give their bank details.)

### Stimuli

**Fig. 1.**
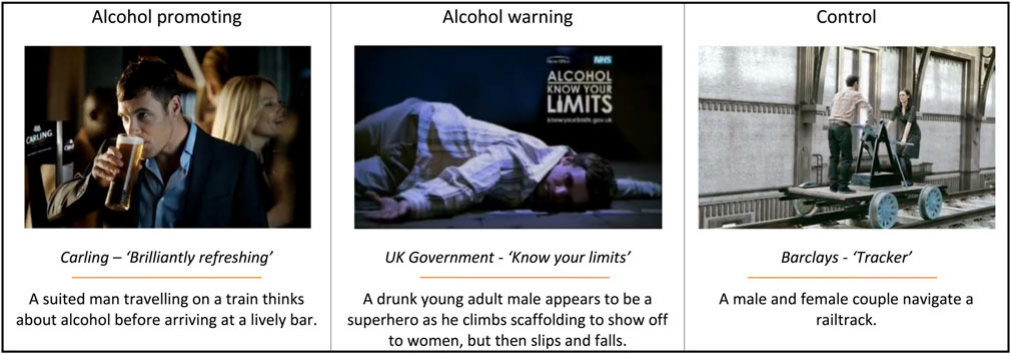
Example advertisements used in study.

Sixteen advertisements were used in total: four alcohol promoting advertisements, four alcohol warning advertisements, four control advertisements for banking products, and four filler advertisements for non-alcohol-related products. Figure 1 presents examples of the advertisements shown. Full descriptions of each advertisement are provided in Supplementary Table S1.

Alcohol promoting, control and filler advertisements were selected from an initial corpus of advertisements selected from UK television and cinema broadcasts over the 5 years prior to the study. Criteria for selecting advertisements were that they were for popular products and were relevant to the target sample. Advertisements were independently ranked for suitability by four authors according to potential appeal, how prominently alcohol was featured, and whether there was potential ambiguity regarding the message content. Two authors independently rated advertisements for their likely target audience using four categories: age, gender, level of education and ethnicity.

Alcohol promoting advertisements for four types of alcohol products (lager, wine, cider and alcopop) were selected. Three of the brands selected (‘Carling’, ‘Smirnoff’, ‘Jacob's Creek’) are in the top 20 alcohol brands by off-trade value in Great Britain (The Grocer, 2014). The advertisements for these brands were respectively targeted towards adult males aged 21–30, adults aged 18–30 of any gender, and adults aged 25–40 of any gender. A fourth (‘WKD’) was selected for its potential appeal to younger participants. The inferred target audience of this advertisement was males aged 18–25.

Alcohol warning advertisements were selected from the UK Department of Health *‘Know Your Limits’* campaign from 2006, which was targeted at 18- to 24-year-old males and females with the aim of reducing heavy episodic drinking (UK Home Office, no date), and from the ‘Change 4 Life’ campaign from 2009, targeted at older adults who drink frequently. These were selected as they are the most recent examples of high profile alcohol use warning message campaigns in the UK.

### Measures

#### Outcome measures

##### Implicit attitudes

Positive and negative implicit attitudes towards alcohol were assessed using two single target unipolar Implicit Association Tests (IAT; Jajodia and Earleywine, 2003; Houben and Wiers, 2008) adapted to assess unipolar attitudes (e.g. positive-neutral and negative-neutral). Category labels consisted of target stimuli (six alcohol-related words: *Wine, Beer, Rum, Whisky, Vodka and Cider*) and attribute stimuli (six positive [*Smiling, Pleasure, Happy, Friendly, Cheerful, Loving*], six negative [*Horrible, Angry, Destroy, Brutal, Tragic, Poison*] or six neutral [*Average, Daily, General, Normal, Usual & Everyday*] words). Exemplars were selected from stimuli used in previous IAT studies.

Shorter average response latencies for the categorization of target exemplars combined with one attribute (e.g. alcohol + positive) relative to the other (e.g. alcohol + neutral) represents a greater implicit attitude towards the former. A measure of effect size (*D*), defined as the average difference (in response latency) *between* the combined categories (e.g. alcohol + positive & alcohol + neutral), divided by the inclusive standard deviation of participants' response latencies *across* the combined categories (Andrews *et al.*, 2010) was calculated as the outcome variable.

##### Explicit attitudes

Explicit attitudes towards alcohol were assessed using 7-point rating scales adapted from a previously used measure (Houben *et al.*, 2010). Two items asked participants how much they considered drinking alcohol to be *Very pleasant – Very unpleasant*, and *Very good – Very bad*. The mean score on the two items was used, with higher scores reflecting more positive explicit attitudes to alcohol. Cronbach's alpha was 0.62.

To reduce demand characteristics, questions regarding explicit attitudes were adapted to reflect the control advertisements (banking), e.g. *I consider my bank to be*: *Very pleasant – Very unpleasant*. Each of these questions used the same response format as their equivalent alcohol-related question.

##### Voucher choice

A choice of alcohol-related voucher to be redeemed in pubs (either ‘Wetherspoons’ or ‘Pubtokens’) or non-alcohol-related voucher to be redeemed in coffee shops (‘Starbucks’ or ‘Costa’) to the value of £5 was offered at the end of the study as additional compensation for participation. This was coded as a dichotomous measure of choice between an alcoholic or non-alcoholic voucher. Such an outcome variable has been used in previous research (Hollands *et al.*, 2011). To prevent alcohol consumption, participants did not receive either voucher, instead receiving the equivalent amount as credit through the research agency.

#### Additional measures

##### Socioeconomic status

As a measure of individual-level socioeconomic status, participants provided their highest level of educational qualification attained, coded onto a five point scale ranging from *0—No qualifications* to *4—Degree or higher*. As a measure of neighbourhood-level socioeconomic status, participants' postcodes were used to derive Index of Multiple Deprivation (IMD; Department for Communities and Local Government, 2010) scores. These scores combine a number of indicators of economic, social, and housing issues into a single deprivation score for each area in England.

##### Alcohol consumption

Past week alcohol consumption was assessed with the following question, derived from a previously used measure (Banerjee *et al.*, 2010): ‘Over the past seven days, how many of each of the following drinks have you drunk? Please think about all drinks you have had, either at home or when you were out.’ Response options provided were: pints of draught beer, lager or cider; bottles of draught beer, lager or cider; large, small, or standard glasses of wine; 25 ml measures of spirits or liqueurs; bottles of pre-mixed drinks; glasses of sherry or port. A continuous score of the number of alcohol units consumed in the past week was calculated from responses to this item. We use the relative term ‘heavier drinkers’ to refer to participants who scored over one standard deviation above the mean in this sample. These participants reported consuming at least 31 units during the past week, an amount that exceeds the Department of Health's (2013) guidelines for ‘lower risk’ drinking (3–4 units per day for males and 2–3 units for females) in the UK, and corresponds to the Centres for Disease Control and Prevention (2014) definition of heavy drinking (consuming 15 or more alcoholic drinks per week for males and 8 drinks or more for females) in the USA. To assess typicality of past week consumption participants were asked ‘Would you say that over the past week, you have drunk…’ with *less/the same amount/more than usual* as the response options.

##### Inhibitory control

The Stop Signal Task (Logan and Cowan, 1984) was used to assess inhibitory control. This task requires participants to rapidly report the identity of a stimulus across trials but to withhold responding on presentation of a specific ‘stop’ stimulus. A greater stop signal reaction time indicates poorer inhibitory control.

##### Advertisement and brand familiarity

Participants' familiarity with advertisements and brands was assessed to ensure similarity of previous product exposure across conditions. Screenshots of each advertisement were presented along with the questions ‘How familiar are you with the advert shown above?’ and ‘How familiar are you with the brand shown in the advert above?’ Seven point visual analogue scales anchored with the terms *Very familiar* and *Very unfamiliar* were presented below each question. Mean familiarity scores for adverts and brands were calculated to create a single score for each with higher scores reflecting increased familiarity.

### Data analysis

Data were analysed in IBM SPSS version 21. One-way ANOVAs were used to examine group differences on demographic variables, alcohol consumption, baseline implicit and explicit attitudes, inhibitory control, brand familiarity, and advertisement familiarity. Attitude change scores were calculated by subtracting post-exposure attitude scores from pre-exposure scores, with positive change scores indicating attitude increases (either reflecting more positive or more negative attitudes). Three multiple linear regressions were then conducted with each change score (positive implicit, negative implicit, and explicit attitudes) as dependent variables. Independent variables were entered in two steps. At step 1, dummy coded variables representing experimental condition (alcohol promoting advertisements and alcohol warning advertisements, each with non-alcohol advertisements as the referent) were entered along with age, gender, education, and past week alcohol unit consumption. At step 2, the product terms of dummy coded condition by education, and condition by alcohol consumption were added to test for interactions. Binary logistic regression was used to test whether condition influenced the choice of alcohol versus non-alcohol voucher. Variables were entered as for linear regressions.

## Results

### Sample characteristics

Demographic characteristics of participants are presented in Table 1. There were no significant between-group differences in these characteristics. Due to a high proportion of degree-educated participants (53%), the education variable was dichotomized into degree-educated and non-degree-educated. No significant group differences were found on baseline implicit or explicit alcohol attitudes, inhibitory control, or brand or advertisement familiarity. The majority of participants (75.6%) reported that their past week alcohol consumption was ‘about the same’ as usual. Five participants were outliers on past week alcohol unit consumption (*z* > 3.29) so were not included in the analysis.

**Table 1.** Demographic characteristics, alcohol consumption, inhibitory control and advert/brand familiarity

	Total	Group		
		Alcohol promoting advertisements	Alcohol warning advertisements	Advertisements unrelated to alcohol
*N*	373	125	129	119
Age	*M* = 28.03 (SD = 5.64)	27.29 (5.72)	28.36 (5.55)	28.44 (5.62)
Gender				
Male	151	49	49	53
Female	222	76	80	66
Highest educational qualification				
No qualification	7	0	5	2
<5 GCSEs/NVQ Level 1	20	5	9	6
5 or more GCSEs/NVQ Level 2	54	17	14	23
A-Levels/NVQ Level 3/Apprenticeship	92	42	29	21
Degree/NVQ Level 4/Professional qualification	194	59	71	64
Ethnicity				
White British	315	99	114	102
White Irish	7	3	2	2
White other	21	9	7	5
Mixed—White and Black African	2	2	0	0
Mixed—White and Asian	5	3	1	1
Mixed other	2	0	1	1
Indian	4	2	1	1
Pakistani	4	1	1	2
Other Asian/Asian British	3	1	1	1
Black/Black British	4	2	0	2
Chinese	6	3	1	2
Index of multiple deprivation	*M* = 21.08 (SD = 15.07)	20.28 (14.07)	21.85 (16.01)	21.12 (15.17)
Past week alcohol consumption (units)	16.02 (15.06)	15.73 (14.20)	16.04 (14.85)	16.32 (16.25)
Stop signal reaction time	272.57 (80.27)	272.02 (70.35)	276.02 (83.44)	269.22 (86.41)
Brand familiarity	5.33 (1.12)	5.46 (1.05)	5.15 (1.12)	5.40 (1.19)
Advertisement familiarity	4.47 (1.34)	4.24 (1.32)	4.54 (1.39)	4.64 (1.29)

### Experimental effects

Table 2 presents mean scores by group on attitudes at baseline and post-exposure, mean attitude changes, and the proportion of participants in each group selecting the alcohol or non-alcohol voucher. Results from multiple linear regression analyses are presented in Table 3.

**Table 2.** Mean (SD) explicit and implicit attitudes pre- and post-advertisement exposure, mean changes, and amount of voucher selections by group

	Total	Group		
		Alcohol promoting advertisements	Alcohol warning advertisements	Advertisements unrelated to alcohol
Positive implicit attitudes				
Baseline	0.02 (.26)	0.05 (0.25)	−0.002 (0.25)	0.02 (0.28)
Post-exposure	−0.004 (.26)	0.01 (0.28)	0.004 (0.24)	−0.03 (0.26)
Change	−0.03 (.36)	−0.04 (0.37)	0.01 (0.34)	−0.05 (0.39)
Negative implicit attitudes				
Baseline	0.10 (.27)	0.10 (0.28)	0.10 (0.24)	0.10 (0.30)
Post-exposure	−0.02 (.32)	0.02 (0.33)	−0.01 (0.31)	−0.05 (0.33)
Change	−0.12 (.41)	−0.08 (0.41)	−0.11 (0.40)	−0.15 (0.42)
Explicit attitudes				
Baseline	5.10 (.1.06)	5.01 (1.09)	5.10 (0.95)	5.20 (1.13)
Post-exposure	5.06 (1.04)	5.12 (1.05)	4.90 (1.06)	5.16 (1.00)
Change	−0.43 (.85)	0.12 (0.85)	−0.21 (0.83)	−0.03 (0.87)
Voucher choice				
Alcohol-related voucher	114	38	40	36
Non-alcohol-related voucher	259	87	89	83

**Table 3.** Multiple regression analyses with attitude change scores as dependent variables

	Positive implicit attitudes				Negative implicit attitudes				Explicit attitudes			
	*b*	SE	*Β*	*t*	*b*	SE	*β*	*t*	*b*	SE	*β*	*t*
Step 1												
Intercept	−0.042	0.045		−0.95	−0.097	0.050	−1.93		−0.180	0.101		−1.78
Condition: Alcohol promoting advertisements	0.001	0.048	−0.002	−0.03	0.062	0.054	0.070	1.14	0.153	0.109	0.086	1.41
Condition: Alcohol warning advertisements	0.049	0.047	0.064	1.04	0.043	0.053	0.050	0.81	−0.163	0.108	−0.092	−1.52
Age	0.000	0.004	−0.003	−0.06	0.004	0.004	0.059	1.09	−0.011	0.008	−0.074	−1.38
Gender	0.071	0.042	0.095	1.71	0.003	0.047	0.004	0.07	0.121	0.094	0.070	1.28
Education	−0.055	0.039	−0.075	−1.41	−0.105	0.044	−0.127	−2.37*	0.197	0.089	0.117	2.21*
Past week alcohol consumption	0.000	0.001	−0.014	−0.25	0.001	0.001	0.039	0.71	0.003	0.003	0.049	0.91
	*R*^2^ = 0.02				*R*^2^ = 0.02				*R*^2^ = 0.05**			
Step 2												
Intercept	−0.057	0.054		−1.06	−0.070	0.060		−1.17	−0.093	0.122		−0.76
Condition: Alcohol promoting advertisements	0.010	0.070	0.013	0.15	0.003	0.078	0.003	0.04	−0.031	0.158	−0.017	−0.20
Condition: Alcohol warning advertisements	0.075	0.071	0.097	1.06	0.037	0.079	0.043	0.47	−0.243	0.160	−0.138	−1.52
Age	0.000	0.004	−0.006	−0.11	0.004	0.004	0.060	1.11	−0.012	0.008	−0.081	−1.51
Gender	0.075	0.042	0.100	1.81	−0.002	0.047	−0.002	−0.04	0.136	0.094	0.079	1.44
Education	−0.027	0.069	−0.037	−0.39	−0.155	0.077	−0.187	−2.01*	0.035	0.157	0.021	0.22
Past week alcohol consumption	−0.004	0.002	−0.156	−1.76	0.007	0.002	0.248	2.82**	−0.003	0.005	−0.048	−0.55
Alcohol promoting*education	−0.022	0.096	−0.022	−0.22	0.119	0.108	0.106	1.11	0.350	0.219	0.153	1.60
Alcohol warning*education	−0.050	0.096	−0.054	−0.52	0.018	0.107	0.017	0.17	0.140	0.216	0.065	0.65
Alcohol promoting*consumption	0.006	0.003	0.145	2.05*	−0.009	0.004	−0.171	−2.23*	0.007	0.007	0.064	0.091
Alcohol warning*consumption	0.004	0.003	0.106	1.45	−0.009	0.003	−0.192	−2.66**	0.011	0.007	0.111	1.54
	*R*^2^ = 0.03				*R*^2^ = 0.05*				*R*^2^ = 0.06*			

Note: Condition is dummy coded with control condition (advertisements unrelated to alcohol) as the referent.

**P < 0.01. *P < 0.05.

#### Implicit attitudes

**Fig. 2.**
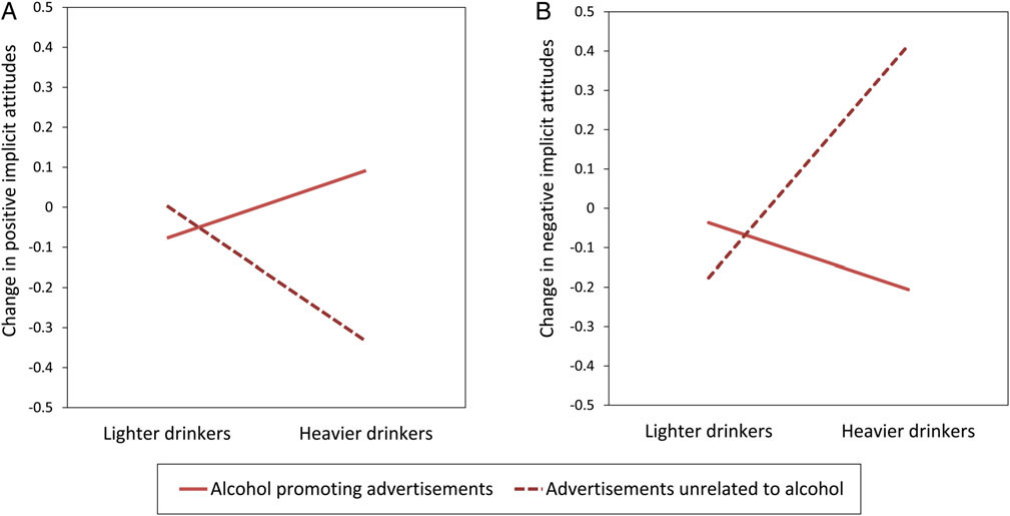
Changes in (**A**) positive implicit attitudes, and (**B**) negative implicit attitudes following exposure to alcohol promoting advertisements and advertisements unrelated to alcohol by heaviness of recent alcohol consumption.

There were no main effects of condition on change in positive implicit attitudes. There was a significant interaction between condition and alcohol consumption (*P* = 0.04): viewing alcohol promoting advertisements (compared to viewing advertisements unrelated to alcohol) increased positive implicit attitudes in heavier drinkers (those scoring +1 standard deviation on the continuous measure of alcohol consumption), but not in lighter drinkers (Figure 2A).

**Fig. 3.**
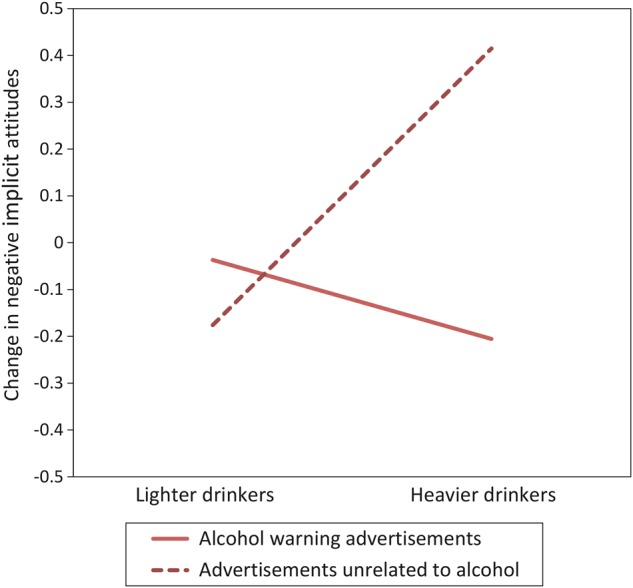
Changes in negative implicit attitudes following exposure to alcohol warning advertisements and advertisements unrelated to alcohol by heaviness of recent alcohol consumption.

There were no main effects of condition on change in negative implicit attitudes. Two significant conditions by alcohol consumption interactions were found: viewing alcohol promoting advertisements (compared to viewing advertisements unrelated to alcohol) decreased negative implicit attitudes in heavier drinkers, but not in lighter drinkers (*P* = 0.02; Figure 2B). Viewing alcohol warning advertisements (compared to viewing advertisements unrelated to alcohol) also decreased heavier drinkers' negative implicit attitudes towards alcohol (*P* = 0.01) (Fig. 3).

#### Explicit attitudes

There were no main effects of condition on change in explicit attitudes. No significant interactions were found.

#### Voucher choice

The majority of participants selected the non-alcohol voucher. Logistic regression analysis indicated no main effects of condition on voucher choice. Planned follow-up mediation and moderated-mediation analyses were not conducted as hypotheses relating to these analyses were conditional on a significant main effect of condition on voucher choice. To assess whether changes in implicit and explicit attitudes were associated with voucher choice, we calculated point-biserial correlations between change scores and choice for the entire sample and for each experimental group. No significant correlations were observed.

## DISCUSSION

This study investigated whether alcohol promoting and alcohol warning advertisements influence attitudes towards alcohol and alcohol seeking behaviour. We found no main effects on these outcomes. There were, however, significant interactions with alcohol consumption: in heavier drinkers, viewing alcohol promoting advertisements increased positive implicit attitudes and decreased negative implicit attitudes towards alcohol. This result is consistent with evidence that exposure to alcohol advertisements leads to increased alcohol consumption immediately following exposure in heavier, but not lighter, drinkers (Koordeman *et al.*, 2011a). Alcohol promoting advertisements may be more salient to heavier drinkers (Field and Cox, 2008), and more likely to trigger positive emotions and memories related to alcohol use. Incentive sensitization theory (Robinson and Berridge, 2001) proposes that greater use of alcohol may lead to alcohol-associated stimuli taking on heightened motivational significance, producing increased desire for alcohol when viewed. The alcohol industry argues that advertising of alcohol products is designed only to promote brand switching (International Centre for Alcohol Policies, 2003). Our data suggest that such advertising also promotes positive implicit attitudes towards alcohol in heavier drinkers. Given evidence that implicit attitudes towards alcohol predict subsequent consumption (Reich *et al.*, 2011), one possible consequence of prevalent alcohol advertising is to reinforce attitudes that make it difficult for heavier drinkers to reduce their alcohol intake.

Viewing alcohol warning advertisements reduced negative implicit attitudes in heavier drinkers. This counterintuitive finding may be evidence of a well-documented ‘boomerang’ effect, whereby messages that attempt to discourage a certain behaviour actually make the behaviour more likely (Ringold, 2002). Such an effect has been observed in studies looking at counter-advertising of alcohol and other substances (Bensley and Wu, 1991; Snyder and Blood, 1992; Czyzewska and Ginsburg, 2007). Heavier drinkers who viewed alcohol warning advertisements may have experienced reactance (Brehm, 1966), implicitly disagreeing with these advertisements to minimize dissonance (Dillard and Shen, 2005). Alternatively, it is possible that heavier drinkers' negative implicit attitudes became less negative due to a sensitization effect of viewing words and images related to alcohol, rather than the specific content of the alcohol warning advertisements. The methods employed do not enable us to clarify the psychological processes that underpin this effect. However, the possibility that alcohol warning advertisements have iatrogenic effects on a population that is a key target for their message warrants further investigation.

Viewing alcohol promoting or alcohol warning advertisements did not change explicit attitudes compared to viewing non-alcohol advertisements, consistent with two experimental studies that have investigated effects of alcohol advertising on explicit attitudes previously (Slater *et al.*, 1996; Goodall and Slater, 2010). We found no evidence that heavier drinkers experienced greater changes in explicit attitudes than lighter drinkers following advertisement exposure, suggesting that cognitive responses to alcohol-related advertising in heavier drinkers may occur only at a less conscious, implicit level. Additionally, we found no evidence of moderation of effects by level of education, indicating that differences in socioeconomic position did not influence cognitive responses to alcohol-related advertising in this sample.

The type of advertisement viewed had no effect on our index of alcohol seeking behaviour. This finding may be a consequence of a choice that offered future, not instant, alcohol consumption. Previous studies that have found an effect of alcohol promoting advertising on alcohol consumption have offered the immediate opportunity to consume alcohol during the experiment (Engels *et al.*, 2009; Koordeman *et al.*, 2011a,b), encouraging impulsive alcohol seeking. In contrast, the presentation of a voucher choice may have triggered deliberative cognitions such as considering an outlet where vouchers might be accepted and planning an appropriate time to use them. Such deliberation may have attenuated any impulsive motivations to use alcohol (Hofmann *et al.*, 2008). In future tests of the hypothesis that advertising influences behaviour via automatic or impulsive processes, researchers should aim to minimize the opportunity for reflection before behaviour by providing an opportunity for immediate consumption.

### Strengths and limitations

Strengths of the current study include using participants that were not recruited from a student population, assessment of alcohol-related attitudes before and after advertisement exposure, and consideration of individual-level factors as potential effect modifiers. The study is the first, to our knowledge, to assess the impact of alcohol warning advertisements explicitly aimed at reducing alcohol consumption and increasing negative attitudes towards excessive consumption. However, a number of limitations affect the interpretation of our findings. We did not assess participants' prior exposure to more general alcohol marketing or media. The association between alcohol promoting advertisements and brand-specific consumption has been shown to diminish at heightened levels of exposure (Ross *et al.*, 2014). Over half of the sample was educated to degree level, and effects of advertising may differ in less educated individuals. The age range of our sample was quite broad, such that any effects of advertisements targeted at individuals of specific demographic characteristics may have been attenuated in participants who were not the target audience. Finally, the alcohol promoting advertisements presented were for four different types of alcoholic beverage. Combining advertisement presentation in this way did not allow us to analyse the effects of specific advertisements.

## CONCLUSION

Our findings suggest that alcohol promoting advertising may have a unique cognitive impact on heavier drinkers, causing an increase in positive associative thoughts about alcohol. Alcohol warning advertisements may cause paradoxical effects in heavier drinkers, leading to a reduction of negative associative thoughts. Further research is needed to test whether either of these effects leads to greater alcohol consumption.

## SUPPLEMENTARY MATERIAL

Supplementary Material is available at *Alcohol and Alcoholism* online.

## FUNDING

This work was jointly supported by the National Institute for Health Research School for Public Health Research, and by the Department of Health Policy Research Program [Policy Research Unit in Behaviour and Health (PR-UN-0409-10109)]. The funding bodies had no role in the study design, data collection and analysis, decision to publish, or preparation of the manuscript. Funding to pay the Open Access publication charges for this article was provided by the National Institute for Health Research School for Public Health Research and by the Department of Health Policy Research Program.

## CONFLICT OF INTEREST STATEMENT

None declared.
